# Dynamic indices of preload in postcardiac surgery patients by pulse power analysis

**DOI:** 10.1186/cc9474

**Published:** 2011-03-11

**Authors:** E Barbon, F Caliandro, J Kamdar, M Puntis, H Meeran, A Rhodes, A Dewhurst, M Cecconi

**Affiliations:** 1 St Georges Hospital, London, UK

## Introduction

The ability to predict fluid responsiveness during the perioperative period is important in order to minimize the risk of hypovolemia and fluid overload. We studied the ability of dynamic indices [1] such as pulse pressure variation (PPV) and stroke volume variation (SVV) measured with the LiDCO™*rapid *to predict the response in stroke volume (SV) after a fluid challenge (FC).

## Methods

This was a prospective observational study of FCs (250 ml colloid given in less than 5 minutes) in the immediate postoperative period in cardiac surgery patients. A positive response to a FC was defined as an increase in SV >10% measured with LiDCO™*rapid*. FCs were repeated according to the unit protocol. PPV and SVV were recorded before FC, together with static haemodynamic measurements: mean arterial pressure (MAP), central venous pressure (CVP) and heart rate (HR). Receiving operator characteristic (ROC) analysis was performed in order to identify haemodynamic variables suitable to predict fluid responsiveness.

## Results

Sixteen patients were enrolled; five females, 11 males, age 70 (± 11) years, weight 82 (± 13) kg, height 167 (± 10) cm. Of the 16 patients, seven (44%) were fluid responders to the first FC. A total number of 47 FCs were given. There were no differences in HR, CVP and MAP between responders and nonresponders. PPV and SVV were significantly different between responders and nonresponders. Areas under the curve for ROC curves were: for PPV 0.76 (0.61 to 0.92), *P *= 0.003, and for SVV 0.80 (0.67 to 0.93), *P *= 0.0006. The best cut-off values (sensitivity and specificity) to predict a SV increase >10% after FC were: PPV >13.5% (79%, 72%), and SVV >10.5% (84%, 68%).

## Conclusions

Dynamic indices measured by LiDCO™*rapid *have a high sensitivity and specificity in predicting fluid responsiveness in fully sedated and mechanically ventilated patients postcardiac surgery.
